# KIF6 gene as a pharmacogenetic marker for lipid-lowering effect in statin treatment

**DOI:** 10.1371/journal.pone.0205430

**Published:** 2018-10-10

**Authors:** Cristina Ruiz-Iruela, Ariadna Padró-Miquel, Xavier Pintó-Sala, Neus Baena-Díez, Assumpta Caixàs-Pedragós, Roser Güell-Miró, Rosa Navarro-Badal, Xavier Jusmet-Miguel, Pilar Calmarza, José Luis Puzo-Foncilla, Pedro Alía-Ramos, Beatriz Candás-Estébanez

**Affiliations:** 1 Clinical laboratory, IDIBELL-Hospital Universitari de Bellvitge, L'Hospitalet de Llobregat, Barcelona, Spain; 2 Cardiovascular unit, IDIBELL-Hospital Universitari de Bellvitge, L'Hospitalet de Llobregat, Barcelona, Spain; 3 Genetics laboratory, Corporació Sanitari Parc Tauli, Sabadell, Barcelona, Spain; 4 Endocrinology department, Corporació Sanitaria Parc Taulí, Sabadell, Barcelona, Spain; 5 Hospitalet Clinical laboratory, Centre Atenció Primària Just Oliveras, L’Hospitalet de Llobregat, Barcelona, Spain; 6 Family medicine, Centre Atenció Primària Just Oliveras, L’Hospitalet de Llobregat, Barcelona, Spain; 7 Clinical laboratory, Hospital Miguel Servet, Zaragoza, Spain; Beijing Key Laboratory of Diabetes Prevention and Research, CHINA

## Abstract

**Introduction:**

The therapeutic response to statins has a high interindividual variability with respect to reductions in plasma LDL-cholesterol (c-LDL) and increases in HDL cholesterol (c-HDL). Many studies suggest that there is a relationship between the rs20455 KIF6 gene variant (c.2155T> C, Trp719Arg) and a lower risk of cardiovascular disease in patients being treated with statins.

**Aim:**

The aim of this study was to investigate whether or not the c.2155T> C KIF6 gene variant modulates the hypercholesteremic effects of treatment with simvastatin, atorvastatin, or rosuvastatin.

**Materials and methods:**

This was a prospective, observational and multicenter study. Three hundred and forty-four patients who had not undergone prior lipid-lowering treatment were recruited. Simvastatin, atorvastatin or rosuvastatin were administered. Lipid profiles and multiple clinical and biochemical variables were assessed before and after treatment.

**Results:**

The c.2155T> C variant of the KIF6 gene was shown to influence physiological responses to treatment with simvastatin and atorvastatin. Patients who were homozygous for the c.2155T> C variant (CC genotype, ArgArg) had a 7.0% smaller reduction of LDL cholesterol levels (p = 0.015) in response to hypolipidemic treatment compared to patients with the TT (TrpTrp) or CT (TrpArg) genotype. After pharmacological treatment with rosuvastatin, patients carrying the genetic variant had an increase in c-HDL that was 21.9% lower compared to patients who did not carry the variant (p = 0.008).

**Conclusion:**

Being a carrier of the c.2155T> C variant of the KIF6 gene negatively impacts patient responses to simvastatin, atorvastatin or rosuvastatin in terms of lipid lowering effect. Increasing the intensity of hypolipidemic therapy may be advisable for patients who are positive for the c.2155T> C variant.

## Introduction

Statins are drugs that specifically inhibit 3-hydroxy-3-methyl-glutaril-CoA reductase, a rate limiting enzyme of the pathway responsible for the synthesis of endogenous cholesterol. Among the lipid-lowering drugs, statins are the ones most often employed to prevent atherosclerosis [1]. In 2015, simvastatin and atorvastin were the third and fifth most prescribed generic drugs in Spain, respectively [2]. However, the therapeutic response to these drugs has a high interindividual variability: the reduction in plasma LDL-cholesterol (c-LDL) resulting from drug intake ranges from 20% to 60%. A similar variability is observed with respect to increases in plasma HDL cholesterol (c-HDL) [3]. The impact genetic variants may have on the effectiveness of statins is currently being investigated [4, 5, 6], with the aim of developing personalized and cost-effective medications.

Many studies have described a relationship between the rs20455 variant of the KIF6 gene (c.2155T> C) and differences in the response to treatment with statins. The c.2155T> C variant is a missense mutation that leads to substitution of an arginine for a tryptophan at position 719 of the KIF6 protein (NP_659464.3: p. Trp719Arg).

KIF6 is a member of the superfamily of kinesins, which are proteins that mediate the intracellular transport of organelles, complex proteins, and mRNAs. Those who carry variant C are at a higher risk of major cardiovascular events (MACE) if they do not receive hypolipidemic treatment [7]. Paradoxically, this allele is also a predictor of better outcomes with statin-based treatments [8, 9]. Specifically, variant C-carriers have been found to be less likely to suffer MACE when treated with pravastatin [10, 11, 12] or atorvastatin [9, 13] than their counterparts carrying the TT (TrpTrp) genotype. However, some reports also suggest that the association of this variant both with MACE and with the response to statins might not exist [14, 15, 16, 17]. Importantly, this SNP is highly prevalent in Europe: 37% of Europeans are carriers [18].

In light of these facts, it is important to clarify what influence this polymorphism might have on the efficacy of statin therapies most commonly employed in our clinical practice, such as atorvastatin, simvastatin or rosuvastatin. Until now, most published studies have focused only on the association between this variant and a lower risk of cardiovascular episodes. However, the effects of statins should be studied more directly. Changes in patient lipid profiles should be measured after treatment begins. This type of study better reflects the realities of clinical practice, in which medical decisions about changes in statin therapy are based primarily on the results of lipid profiling [19].

## Objective

The objective of this study was to examine how the c.2155T> C variant of the KIF6 gene influences the hypolipidemic and hypocholesterolemic responses to simvastatin, atorvastatin, and rosuvastatin. Responses were quantified by measuring differences in the plasma concentration of c-LDL, Non-HDL cholesterol (c-Non-HDL), and c-HDL after treatment.

## Materials and methods

### Population selection

The c.2155T> C variant of the KIF6 gene was chosen as the object of study as part of an ongoing line of research into the genetic factors that influence patient responses to statins. Of particular interest were genetic variants of genes that play a role in the pharmacodynamics and metabolism of these drugs. This was a prospective, observational, and multicenter study. Non-medicated patients attending several Spanish Primary Health Care Units and Hospital Cardiovascular Risk Units with high-LDL concentration were managed as usual by their physicians. As the design of this study was observational, statin drugs, if necessary, were prescribed in conditions of normal medical practice and according solely to the patients’ physicians criteria. The patients who were excluded from the study were those who: 1) had chronic liver disease; 2) had familial hypercholesterolemia due to mutations in their LDLR, APOB, LDLRAP1 or PSCK9 genes; 3) suffered dysbetalipoproteinemia; 4) were receiving antidepressant, antiepileptic, immunosuppressants, antiretroviral or other lipid-lowering treatments 5) were suffering autoimmune diseases; 6) had discontinued treatment or were suspected of not adhering to prescriptions, which was verified during the follow-up visit through the interview and the medical criteria 7) had statin intolerance, which is defined as the inability to tolerate a dose of statin required to reduce MACE sufficiently from their baseline risk and could result from different statin related side effects including: muscle symptoms, headache, sleep disorders, dyspepsia, nausea, rash, alopecia, erectile dysfunction, gynecomastia, and/or arthritis [20] 8) had hypothyroidism; 9) were consuming 6 or more drugs (polymedication); 10) were already participating in a clinical trial.

### Procedures and interventions

#### Data collection

Patient demographic and clinical information were collected during initial consultations. No treatments were administered during this consultation; however, lipid metabolism was evaluated. This evaluation included measurements of triglycerides, total cholesterol, c-Non-HDL, c-HDL, and c-LDL. The Friedewald formula was used when triglyceride concentrations did not exceed 2.3 mmoL/L. An identical evaluation of lipid metabolism was made during the final consultations, which occurred approximately 3 months after treatment was initiated.

#### Genetic analysis

DNA extraction from blood samples was performed with the Maxwell 16 Blood DNA Purification Kit (catalog# AS1010) and the Maxwell 16 System Kit (catalog#AS1010) (Promega, Madison, USA). Real-time polymerase chain reaction (RT-PCR) was used to detect the c.2155T> C variant of the KIF6 gene and also the 521T> C variant of the SLCO1B1 gene. The amplification was performed using all-specific Applied Biosystems Taqman probes labelled with fluorochrome in a 7500 Real Time PCR System thermocycler (Applied Biosystems, Foster City, CA, USA).

#### Statistical analysis

The following independent control variables were analyzed: age, sex, diabetes, hypertension, diastolic blood pressure, systolic blood pressure, exercise, intensity of exercise, history of smoking, current tobacco use, history of alcohol consumption, current alcohol consumption, body mass index, initial concentrations of c-LDL, c-Non-HDL and c-HDL, detection of lipoprotein a, prior ischemia, family history of ischemia, and carrying the SLCO1B1 gene variant rs4149056 [21]. Models were also fitted according to dose. Treatment intensity was evaluated qualitatively (low, moderate, and high) according to the clinical practice guide of the American College of Cardiology [22]. The statistically significant control variables (p<0.05) were selected for each model according to a simple linear regression with the lipid concentration variations. An explanatory quantitative multiple linear regression analysis was carried out in order to discern the relationship between the differences in patient c-LDL, c-Non-HDL and c-HDL measurements that were made during the two consultations and detection of the c.2155T > C variant. Each statin treatment group was analyzed in this way.

The relationship between lipid concentration variations and being a carrier of the c.2155T> C variant was assessed using additive models. When significant differences were observed, these were grouped according to the most appropriate model for each case (recessive or dominant).

Statistical analyses were performed with SPSS v.17 (SPSS Inc., Chicago, IL). The level of significance was set at *p*<0.05.

### Ethical and confidentiality issues

Informed consent was received from study participants during their initial consultation. In compliance with regulation #SAS/3470/2009, this study was classified by the Pharmacoepidemiology Division of the Medicines and Health Products Agency of Spain (ref. PR169/14) and approved by the institutional review boards of each participating center: *Comité Ético de Investigación Clínica (CEIC) del Hospital Universitario de Bellvitge*, *CEIC Hospital universitario Miguel Servet* and *CEIC Corporació Sanitària Parc Taulí*.

## Results

### Population selection

From June 2014 to February 2017, 344 patients were recruited prospectively. However, 92 of these were later excluded. Of the excluded, 33 had had prior hypolipidemic treatment, 3 had statin intolerance related with muscle complications (myopathy and rhabdomyolysis), 9did not adhere to the treatment regimen, 20 did not participate in follow-ups, 11 had been diagnosed, through genetic testing, with familial hypercholesterolemia, 2 had hypothyroidism, 1 had an autoimmune disease. Another thirteen were excluded for miscellaneous reasons. Consequently, only the data from 252 of total number patients recruited for the study was analyzed.

### Descriptive statistics

The clinical and demographic data of the patients is found in **Table 1.**

**Table 1:** Patient clinical, demographic, biochemical and treatment characteristics depending on the c.2155T>C (Trp719Arg) KIF6 genotypes.

**Clinical and Demographic Variables**	**TT (TrpTrp)**	**TC (TrpArg)**	**CC (ArgArg)**	**p value**
Sex (% male)	48.1	51.9	57.9	0.553
Age (years)	53 (50 to 55)	54 (51 to 56)	57 (53 to 61)	0.201
Body Mass Index	26.7 (25.8 to27.7)	27.3 (26.5 to 28.0)	27.5 (26.0 to 29.0)	0.574
Tobacco Use (% yes)	37.7	21.7	19.4	0.025*
Personal history of tobacco use (% yes)	71.0	58.5	55.2	0.092
Diabetes mellitus (% yes)	18.8	13.2	24.2	0.267
Current alcohol consumption (% yes)	33.3	24.3	30	0.429
Personal history of alcohol consumption (% yes)	27.1	21.3	27.6	0.621
MACEs (% yes)	13.6	19.8	28.9	0.049*
Family history of MACEs (% yes)	38.6	38.5	35.7	0.960
Exercise (% yes)	55.4	59	65.6	0.616
Intensity of Exercise (none/low/moderate/high) %	33.3/21.6/21.6/23.5	34.6/17.3/17.3/30.9	34.8/8.7/8.7/47.8	0.417
Arterial hypertension (% yes)	27.2	30.5	36.8	0.563
Systolic Blood Pressure (SBP) (mmHg)	129.3 (125.8 to 132.8)	131.5 (128.0 to 135.0)	132.8 (127.7 to 138.0)	0.535
Diastolic Blood pressure (DBP) (mmHg)	79.8 (77.4 to 82.1)	79.0 (76.6 to 81.2)	79.2 (75.2 to 83.1)	0.891
**Biochemical and Treatment Variables**	**TT (TrpTrp)**	**TC (TrpArg)**	**CC (ArgArg)**	**p value**
Lipoprotein A (reference value: 0–0.3 g/L)	0.4 (0.3 to 0.5)	0.5 (0.4 to 0.6)	0.5 (0.2 to 0.7)	0.364
Serum cholesterol; initial (mmol/L)	7.3 (7.1 to 7.6)	7.1 (6.9 to 7.3)	7.2 (6.7 to 7.6)	0.562
Serum cholesterol LDL; initial (mmol/L)	5.1 (4.8 to 5.3)	4.9 (4.7 to 5.1)	4.9 (4.5 to 5.3)	0.627
Serum cholesterol no HDL; initial (mmol/L)	5.8 (5.6 to 6.0)	5.7 (5.5 to 5.9)	5.6 (5.2 to 6.0)	0.650
Serum triglycerides; initial (mmol/L)	1.9 (1.6 to 2.17)	2.1 (1.8 to 2.4)	1.7 (1.4 to 2.1)	0.343
Treatment with atorvastatin (%)	38.3	51.1	42.1	0.338
Treatment with simvastatin (%)	50.6	37.4	42.1	
Treatment with rosuvastatin (%)	11.1	11.5	15.8	
Intensity of Treatment (low/medium/high) %	(12.3/63/24.7)	(11.5/59.5/29)	(10.5/44.7/44.7)	0.271

Continuous variables are expressed as averages and as 95% confidence intervals (CI95%). Categorical variables are expressed in percentages.

* indicates statistical significance.

Simvastatin, atorvastatin, and rosuvastatin were prescribed to 106, 116 and 30 patients, respectively. The genotype frequencies of this sample fulfilled the Hardy-Weinberg equilibrium and their distribution matched those found in the HapMap of Europe [14], (**Table 2**).

**Table 2:** Genotype distribution of the genetic variants investigated, expressed in percentages.

c.2155T>C (Trp719Arg) gene KIF6	Genotype distribution (%)			*p*
**HapMAP**	TT (37.2)	CT (51.3)	CC (11.5)	
**Simvastatin+Atorvastatin+Rosuvastatin**	TT (32.4)	CT (52.4)	CC (15.2)	0.37
	n = 81	n = 131	n = 38	
**Simvastatin**	TT (38.7)	CT (46.2)	CC (15.1)	0.56
	n = 41	n = 49	n = 16	
**Atorvastatin**	TT (28.4)	CT (57.7)	CC (13.7)	0.18
	n = 31	n = 67	n = 16	
**Rosuvastatin**	TT (30.0)	CT (50.0)	CC (20.0)	0.38
	n = 9	n = 15	n = 6	

*p*: probability of statistical significance as calculated using the Chi squared test.

**Fig 1:**
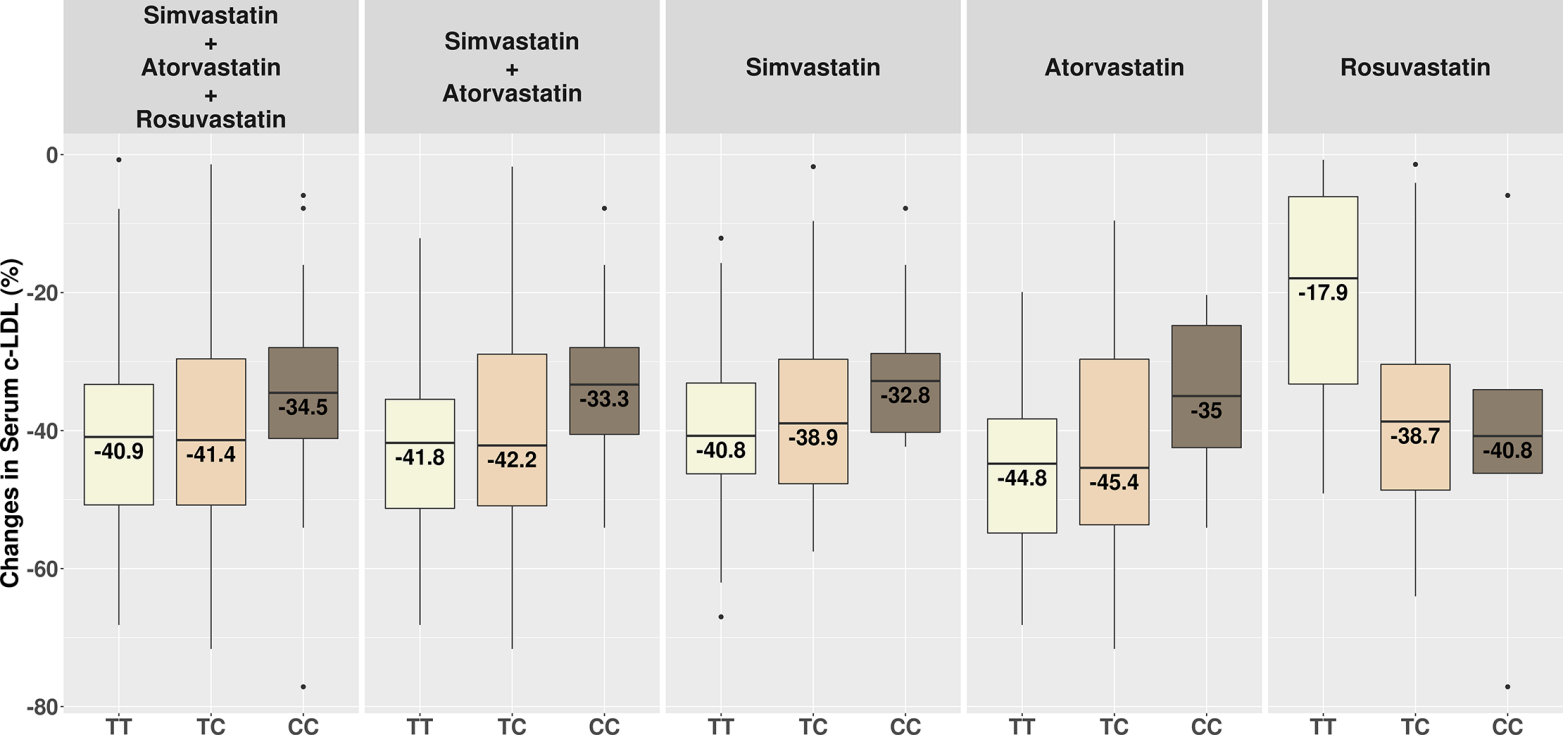
Changes in serum c-LDL after treatment stratified by genotypes. *TT* = homozygous TrpTrp; *TC* = heterozygous TrpArg; *CC* = homozygous ArgArg.

**Fig 2:**
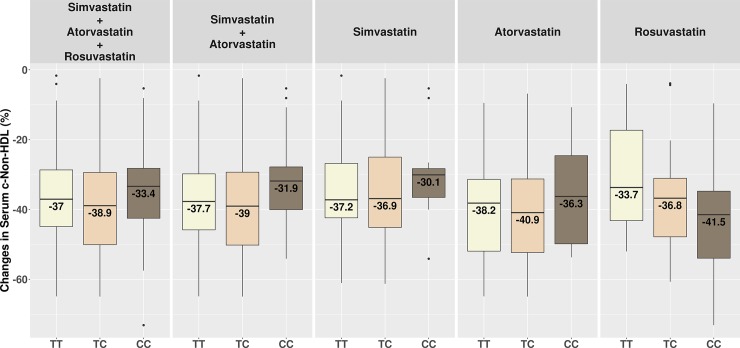
Changes in serum c-Non-HDL after treatment stratified by genotypes. *TT* = homozygous TrpTrp; *TC* = heterozygous TrpArg; *CC* = homozygous ArgArg.

**Fig 3:**
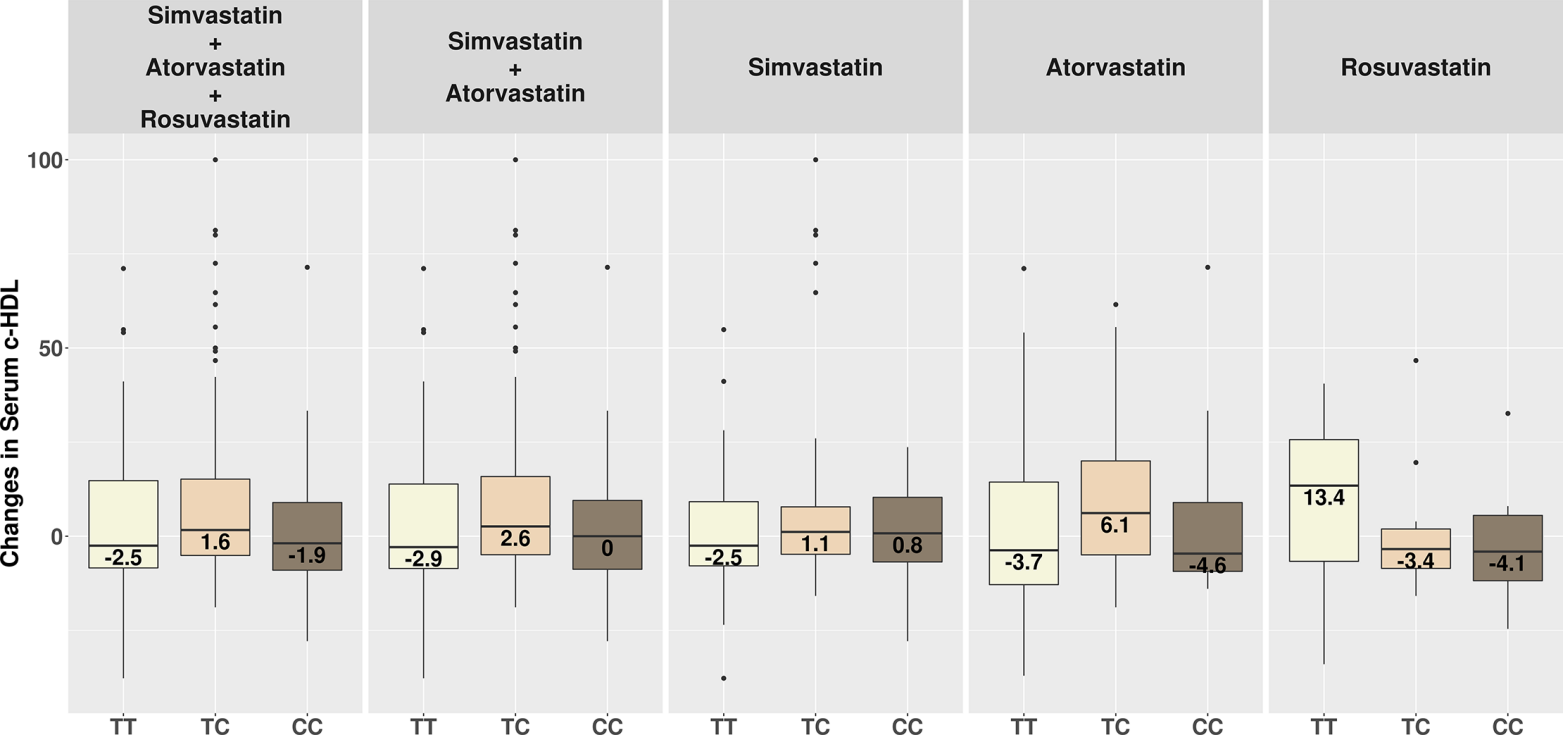
Changes in serum c-HDL after treatment stratified by genotypes. *TT* = homozygous TrpTrp; *TC* = heterozygous TrpArg; *CC* = homozygous ArgArg.

Figs 1, 2 and 3 show changes in serum c-LDL, c-Non-HDL and c-HDL after treatment stratified by genotypes.

Tables 3, 4 and 5 contain the results for each drug as well as the dependent variables.

**Table 3:** Multiple regression analysis that correlates the effect of the variant KIF6 on the LDL cholesterol concentration adjusted by statistically significant covariates.

Changes in Serum LDL cholesterol concentration after treatment; (%)				
Treatment	Model	Variables	*B + CI* (95%)	P
**Simvastatin+Atorvastatin+Rosuvastatin**	*Additive*	**Genotypes: TT, TC, and CC**	2.8 (-0.2 to 5.9)	0.070
		Intensity of qualitative treatment	-4.7 (-8.1 to -1.3)	0.008*
		Initial concentration of c-LDL	-2.4 (-4.3 to -0.6)	0.006*
**Simvastatin+ Atorvastatin**	*Additive*	**Genotypes: TT, TC, and CC**	4.0 (1.0 to 6.8)	0.010*
		Intensity of qualitative treatment	-6.0 (-9.2 to -2.6)	4.1x10-4*
		Initial concentration of c-LDL	-2.4 (-4.2 to -0.6)	0.008*
	*Recessive*	**KIF6 (CC compared to T)**	7.0 (1.3 to 12.6)	0.015*
		Intensity of qualitative treatment	-6.0 (-9.2 to -2.6)	4.1x10-4*
		Initial concentration of c-LDL	-2.4 (-4.2 to -0.6)	0.008*
**Simvastatin**	*Additive*	**Genotypes: TT, TC, and CC**	3.8 (-0.04 to 7.7)	0.053
		Intensity of qualitative treatment	-6.0 (-11.8 to -0.03)	0.039*
**Atorvastatin**	*Additive*	**Genotypes: TT, TC, and CC**	4.4 (-0.2 to 9.1)	0.064
		Intensity of qualitative treatment	-6.6 (-11.7 to -1.5)	0.011*
		Initial concentration of c-LDL	-2.9 (-5.3 a -0.5)	0.016*
**Rosuvastatin**	*Additive*	**Genotypes: TT, TC, and CC**	-7.0 (-20.2 a 6.0)	0.275
		Prior MACE	-24.1 (-4.,7 to -2.5)	0.031*

B+CI (95%) = coefficient B + a 95% confidence interval

* indicates statistical significance.

**Table 4:** Multiple regression analysis that correlates the effect of the variant KIF6 on the Non-HDL cholesterol concentration adjusted by statistically significant covariates.

Changes in Serum Non-HDL Cholesterol concentration after treatment; (%)				
Treatment	Model	Variables	*B + CI* (95%)	P
**Simvastatin+Atorvastatin+Rosuvastatin**	*Additive*	**Genotypes: TT, TC, and CC**	0.8 (-1.7 to 3.4)	0.535
		Intensity of qualitative treatment	-4.4 (-7.2 to -1.5)	0.003*
		Initial concentration of c-Non-HDL	-2.6 (-4.0 to -1.2)	2.6x10-4*
**Simvastatin+ Atorvastatin**	*Additive*	**Genotypes: TT, TC, and CC**	1.7 (-0.9 to 4.4)	0.200
		Intensity of qualitative treatment	-5.3 (-8.2 to 2.3)	4.0x10-4*
		Initial concentration of c-LDL	-2.6 (-4.0 to -1.2)	2.2x10-4*
	*Recessive*	**KIF6 (CC compared to T)**	5.1 (0.1 to 10.1)	0.045*
		Intensity of qualitative treatment	-5.4 (-8.2 to -2.5)	2.8x10-4*
		Initial concentration of c-LDL	-2.6 (-4.0 to -1.2)	2.6x10-4*
**Simvastatin**	*Additive*	**Genotypes: TT, TC, and CC**	1.2 (-2.2 to 4.7)	0.504
		Initial concentration of c-Non-HDL	-3.4 (-5.4 to -1.3)	0.001*
**Atorvastatin**	*Additive*	**Genotypes: TT, TC, and CC**	3.6 (-0.4 to 7.6)	0.070
		Intensity of qualitative treatment	-6.2 (-10.6 to -1.8)	0.006*
		Initial concentration of c-Non-HDL	-2.8 (-4.7 to -1)	0.004*
		Age	-0.3 (-0.5 to 0.1)	0.001*
	*Recessive*	**KIF6 (CC compared to T)**	9.03 (2.1 to 16.5)	0.012*
		Intensity of qualitative treatment	-6.8 (-11.1 to -2.4)	0.003*
		Initial concentration of c-Non-HDL	-2.8 (-4.7 to -1.0)	0.003*
		Age	0.35 (0.15 to 0.54)	0.001*
**Rosuvastatin**	*Additive*	**Genotypes: TT, TC, and CC**	-3.4 (-12.9 to 6.0)	0.463
		Prior MACE	-15.4 (-33-3 to 2.5)	0.089

B+CI (95%) = coefficient B + a 95% confidence interval

* indicates statistical significance.

**Table 5:** Multiple regression analysis that correlates the effect of the variant KIF6 on the HDL cholesterol concentration adjusted by statistically significant covariates.

Change in Serum HDL Cholesterol concentration after treatment; (%)				
Treatment	Model	Variables	*B + CI* (95%)	P
**Simvastatin+Atorvastatin+Rosuvastatin**	*Additive*	**Genotypes: TT, TC, and CC**	0.3 (-3.8 to 4.6)	0.860
		Initial concentration of c-HDL	-26.6 (-33.4 to -19.8)	3.4x10-13*
**Simvastatin+ Atorvastatin**	*Additive*	**Genotypes: TT, TC, and CC**	1.0 (-3.5 to 5.6)	0.661
		Initial concentration of c-HDL	-28.7 (-36.1 to -21.3)	7.1x10-13*
**Simvastatin**	*Additive*	**Genotypes: TT, TC, and CC**	1.4 (-4.8 to7.7)	0.648
		Initial concentration of c-HDL	-31.9 (-42.2 to -21.6)	1.39x10-8*
**Atorvastatin**	*Additive*	**Genotypes: TT, TC, and CC**	0.6 (-6.3 to 7.5)	0.857
		Initial concentration of c-HDL	-25.4 (-36.4 to -14.4)	1.18x10-5*
**Rosuvastatin**	*Additive*	**Genotypes: TT, TC, and CC**	-11.3 (-21.5 to -1.2)	0.030*
		Current tobacco use	-36.3 (-60.1 a -12.5)	0.004*
	*Dominant*	**KIF6 (C compared to TT)**	-21.9 (-37.6 to -6.2)	0.008*
		Current tobacco use	-41.9 (-65.7 to 18.0)	0.001*

B+CI (95%) = coefficient B + a 95% confidence interval

* indicates statistical significance.

Intensity of qualitative treatment and the initial concentration of each lipid were found as significant control variables in the case of atorvastatin, simvastatin and all patients studied together. Concerning atorvastatin and c-Non-HDL variation, the age was also included. Regarding rosuvastatin, prior MACE was found as significant variable for the c-LDL and c-Non-HDL variation, and current tobacco use in the case of c-HDL variation.

Concerning patients that had been treated with simvastatin, an almost statistically significant relationship was observed between being a carrier of the c.2155T> C variant of KIF6 and changes in c-LDL after treatment (p = 0.053). Treatment responses were less pronounced among those patients who were homozygous for the mutation (CC, ArgArg) than among those who were homozygous for the non-mutated gene (TT, TrpTrp). A similar trend was observed with patients who had been treated with atorvastatin. In this case, there was a statistically significant relationship between the c.2155T> C variant of KIF6 and the c-Non-HDL variation after treatment (p = 0.012). There was also a clear tendency for the presence or absence of the variant to be associated with c-LDL variation after treatment (p = 0.06). However, an association between the c.2155T> C variant and c-LDL and c-Non-HDL variation after treatment was not observed in the group of patients treated with rosuvastatin (p = 0.353 and p = 0.454, respectively)

When all 252 recruited patients were included in the statistical analyses and the control variables were adjusted for, a clear tendency for the c.2155T> C variant and the reduction in plasma c-LDL after treatment was found. (Additive model: p = 0.07; B + IC95% = 2.8 (-0.2 to 5.9)). When only taking into account patients who underwent treatment with simvastatin and atorvastatin, this relationship was even more pronounced, and a statistically significant relationship was found: (additive model: p = 0.010; B + IC95% = 4 (1 to 6.8); recessive model: p = 0.015; B + IC95% = 7 (1.3 to 12.6)).

There was also a statistically significant relationship between being a carrier of the variant c.2155T> C and a weaker response to rosuvastatin in comparison to non-carriers, with higher levels of c-HDL being observed after treatment (p = 0.03). However, no statistically significant relation was found between the variant and c-HDL level changes in the simvastatin and atorvastatin treatment groups (p = 0.648 and p = 0.857, respectively).

Based on these results, we can conclude that being a carrier of the c.2155T> C variant of the KIF6 gene negatively impacts patient responses to statin treatments. A less pronounced decrease in c-LDL in the case of simvastatin and atorvastatin and less pronounced increase in c-HDL in the case of rosuvastatin are observed with respect to non-carriers.

## Discussion

Statins are highly effective prophylactics against arteriosclerosis. Nevertheless, a high proportion of patients consuming statins develop some type of cardiovascular disease. These outcomes suggest that genetic factors may influence patient responses to treatment with statins [23]. Numerous genes, such as SLCO1B1, CETP, ABCA, HMGCR, and CYP3A4 are currently being investigated [24, 25] as possible factors affecting statin therapy outcomes. Of these, there has been a particular focus on KIF6 gene and its variant c.2155T> C (Trp719Arg).

A member of the kinesin superfamily, the KIF6 gene encodes the protein KIF6. Kinesins are proteins which mediate the intracellular transport of organelles, complex proteins and mRNAs. This gene is ubiquitously expressed in coronary arteries and other vascular tissue [26].

The effects of the c.2155T> C variant of KIF6 on the ability of statins to reduce the risk of cardiovascular events has been documented, but the results are contradictory. Several studies carried out by Iakouvova et al. [10, 11, 12, 13] concluded that patients that are carriers for the variant (TC (TrpArg) + ​​CC (ArgArg)) are less likely, compared to non-carriers, to suffer from coronary heart disease when treated with pravastatin or atorvastatin. Meanwhile, a meta-analysis by Shiffman et al. [27] concluded that the variant affected responses to pravastatin treatment. Nevertheless, these conclusions are based on data from a single research group and therefore should be confirmed in other studies carried out independently by other groups.

Importantly, this association was not confirmed in other studies of simvastatin [28], rosuvastatin [29] and atorvastatin therapies [30].

In addition, others, such as Chen S. et al [31] have recently observed that patients undergoing statin therapy were at significantly reduced risk for MACE in the case of all three Trp719Arg polymorphisms. Furthermore, subjects carrying (TC) TrpArg or CC+CT (ArgArg+TrpArg) suffered from a greater incidence of MACE when being treated with statins than TT (TrpTrp).

As far as we know, our study is the first attempt to study the relationship between the KIF6 gene variant and the effects of statins on changes on lipid profiles using a prospective and observational design. In addition, the analyses of these effects have been adjusted against control variables. Importantly, normal clinical procedures were reproduced: medical decisions about therapeutic treatments were based primarily on changes in patient lipid profiles.

According to our results, patients homozygous for variant C did not respond as well to treatment with simvastatin and atorvastatin as did the patients who were homozygous or heterozygous for variant T. The same tendency was observed with rosuvastatin when measuring increases in c-HDL levels. Considering these results, it should be noted that while Rosticci et al [32], did not observe a relationship between the KIF6 variant and statin therapy efficacy, this group did observe a relationship between the variant and c-HDL levels.

However, this relationship was not observed for rosuvastatin when measuring changes in c-LDL and c-Non-HDL. This discrepancy could be explained by the fact that there were a low number of rosuvastatin cases included in the study. Rosuvastatin is usually prescribed to high risk populations when other statins have already been found to be insufficient. Therefore, there were fewer of rosuvastatin cases because this prospective and observational study only included individuals who had not undergone prior hypocholesterolemic treatment.

In a study carried out by Angelini et al. [33] it was shown that there was no relationship between the KIF6 variant and the effects of statins on patient lipid profiles. Although an observed trend says that subjects carrying CC (ArgArg) do not respond as well to treatment as TT (TrpTrp) and TC (TrpArg) subjects. However, in contrast to ours, the study of Angelini et al. was retrospective in nature, rather than prospective. In addition, the Angelini et al. study did not adjust for dose intensity or type of statin.

Moreover, the dependent variable was qualitative: the sole criteria it depended on was whether or not a clinical target of c-LDL ≤ 3.4 (130 mg / dL) mmol / L was reached or not [34].

Importantly, our results could be consistent with the latest information from the literature. We found that there was a statistically significant correlation between being a sufferer of MACE and being a carrier of c.2155T> C variant of KIF6 when taking into consideration all patients prior to treatment with statins (p = 0.049). This outcome is consistent with the current literature [35, 36, 37].

In addition, we have demonstrated that, while, c-LDL levels are reduced in all the three genotypes in response to treatment with simvastatin and atorvastatin, c-LDL levels are reduced to a lesser degree in the case of the variant.

Moreover, there is a general consensus that carriers benefit more from statin therapy in terms of a lower risk of suffering from MACE. However, the reason for this relationship may not lie in the fact that c-LDL levels decrease to a greater extent in non-carriers than in carriers.

The mechanism through which KIF6 influences statin therapy outcomes is not well understood. However, our study is an important step to clarifying the relationship between the c.2155T> C variant of KIF6 and responses to statin treatment. If our results can be confirmed, we could hypothesize that the carriers undergoing statin treatment suffer less from MACEs due to the pleiotropic effects of these drugs rather than a reduction in c-LDL. This hypothesis is consistent with the theory posited by Iakoubova et al. [13]. In the PROVE IT-TIMI 22 study, no statistically significant relationship was found between the variant and a reduction of c-LDL levels in response to atorvastatin or pravastatin, however, this group found that carriers benefited significantly more from intensive statin therapy than non-carriers and hypothesized that the mechanism could be the early plaque-stabilizing effect of the intensive treatment regimen, a pleiotropic effect that has been proposed to explain the early benefit from statin therapy that appears not to be due to LDL [38, 39]. However, functional studies of the KIF6 gene will be required to a better understand of the mechanism between c.2155T> C variant and statin treatment.

A key outcome of our study is that we have demonstrated that carriers of variant C have a 7% smaller decrease in c-LDL or c-Non-HDL levels in response to simvastatin or atorvastatin. The fact that the less common variant C is associated with a weaker response to treatment is especially relevant. If this relationship were to be confirmed, patients carrying variant C could be considered candidates for intensive treatment with stronger statins or complementary therapies. Complementary therapies include the drug Ezetimibe, or dietary supplements, such as omega-3.

## Conclusions

The c.2155T> C variant of the KIF6 gene influences patient responses to treatment with simvastatin and atorvastatin. Patients homozygous for the variant (CC, ArgArg) had a smaller decrease in the c-LDL and c-Non-HDL. Therefore, these patients did not respond as well to hypolipidemic therapies as patients who were homozygous TT (TrpTrp) or heterozygous TC (TrpArg). The c.2155T> C variant is associated with a less pronounced increase in c-HDL upon rosuvastatin treatment.

These findings, if confirmed, may have an impact on the type of therapies selected for patients carrying the genetic variant. For example, the intensity of statin therapy could be increased, or complementary therapies, such as Ezetimibe or dietary supplements could be employed to treat carriers.
